# Interaction between Advanced Glycation End Products Formation and Vascular Responses in Femoral and Coronary Arteries from Exercised Diabetic Rats

**DOI:** 10.1371/journal.pone.0053318

**Published:** 2012-12-28

**Authors:** Maria A. Delbin, Ana Paula C. Davel, Gisele Kruger Couto, Gustavo G. de Araújo, Luciana Venturini Rossoni, Edson Antunes, Angelina Zanesco

**Affiliations:** 1 Department of Physical Education, Institute of Bioscience, University of São Paulo State (UNESP), Rio Claro, São Paulo, Brazil; 2 Department of Anatomy, Cellular Biology, Physiology and Biophysics, Institute of Biology, University of Campinas (UNICAMP), Campinas, São Paulo, Brazil; 3 Department of Physiology and Biophysics, Institute of Biomedical Sciences, University of São Paulo (USP), São Paulo, São Paulo, Brazil; 4 Department of Pharmacology, Faculty of Medical Sciences, University of Campinas (UNICAMP), Campinas, São Paulo, Brazil; University of Tor Vergata, Italy

## Abstract

**Background:**

The majority of studies have investigated the effect of exercise training (TR) on vascular responses in diabetic animals (DB), but none evaluated nitric oxide (NO) and advanced glycation end products (AGEs) formation associated with oxidant and antioxidant activities in femoral and coronary arteries from trained diabetic rats. Our hypothesis was that 8-week TR would alter AGEs levels in type 1 diabetic rats ameliorating vascular responsiveness.

**Methodology/Principal Findings:**

Male Wistar rats were divided into control sedentary (C/SD), sedentary diabetic (SD/DB), and trained diabetic (TR/DB). DB was induced by streptozotocin (i.p.: 60 mg/kg). TR was performed for 60 min per day, 5 days/week, during 8 weeks. Concentration-response curves to acetylcholine (ACh), sodium nitroprusside (SNP), phenylephrine (PHE) and tromboxane analog (U46619) were obtained. The protein expressions of eNOS, receptor for AGEs (RAGE), Cu/Zn-SOD and Mn-SOD were analyzed. Tissues NO production and reactive oxygen species (ROS) generation were evaluated. Plasma nitrate/nitrite (NO_x_^−^), superoxide dismutase (SOD), catalase (CAT), thiobarbituric acid reactive substances (TBARS) and N^ε^-(carboxymethyl) lysine (CML, AGE biomarker). A rightward shift in the concentration-response curves to ACh was observed in femoral and coronary arteries from SD/DB that was accompanied by an increase in TBARS and CML levels. Decreased in the eNOS expression, tissues NO production and NO_x_^−^ levels were associated with increased ROS generation. A positive interaction between the beneficial effect of TR on the relaxing responses to ACh and the reduction in TBARS and CML levels were observed without changing in antioxidant activities. The eNOS protein expression, tissues NO production and ROS generation were fully re-established in TR/DB, but plasma NO_x_^−^ levels were partially restored.

**Conclusion:**

Shear stress induced by TR fully restores the eNOS/NO pathway in both preparations from non-treated diabetic rats, however, a massive production of AGEs still affecting relaxing responses possibly involving other endothelium-dependent vasodilator agents, mainly in coronary artery.

## Introduction

Exercise training is positively associated with better prognostic outcomes of certain chronic pathological conditions such as arterial hypertension, dyslipidemia, diabetes mellitus and obesity [1], [2]. Increased nitric oxide (NO) production and/or its bioavailability to the tissues are the hallmark of the health-beneficial effects of exercise training [3], [4]. These effects are mainly associated with an up regulation of Cu/Zn-superoxide dismutase protein expression or a decreased protein expression of NADPH oxidase gp91^phox^ and p47^phox^ subunits resulting in improvement of relaxing response as well as reducing systemic pro-inflammatory mediators [5]–[8].

Type 1 and type 2 diabetes mellitus are cardiometabolic diseases characterized by chronic hyperglycemia which has been associated with severe tissue damage resulting in long-term clinical outcomes such as kidney disease, retinopathy, vascular and neuron defects that result in foot problems and cardiovascular diseases [9]–[12]. Persistent hyperglycemia in both types of diabetes mellitus can activate alternative glucose metabolism pathways that in turn result in the formation of deleterious products derived from protein or lipid structure alterations named advanced glycation end products (AGEs) [13], [14]. The most characterized AGEs compound is N^ε^-(carboxymethyl) lysine (CML) that is generated from the reaction of dicarbonyl products with lysine or arginine functional groups on proteins [15].

In endothelial cells, the interaction of AGEs with their receptor (RAGE) can activate complex signaling pathways causing increased production of pro-inflammatory mediators and generation of reactive oxygen species (ROS) [15]. Indeed, the deleterious effect of AGEs is related to reduction in half-life of NO synthase (eNOS) mRNA through an increased rate of mRNA degradation resulting in reduced eNOS activity [16]. Additionally, AGEs-bound RAGE in the endothelium leads to a massive ROS production through the activation of oxidant enzyme, NADPH oxidase, which increases the superoxide anion (O_2_^−^) inactivating NO bioavailability [17]–[19]. Nuclear factor-kappa B (NF-kB) is also activated by AGEs in diabetic state resulting in transcription of inflammatory factors as well as increased endothelin-1 production. Hence an imbalance between vasodilator and vasoconstrictor agents production as well as increased O_2_^−^ production in endothelium are believed to be as consequence of AGEs production in the diabetic state leading to vascular dysfunction [15].

Although the effects of exercise training in diabetic state have been largely studied in both human [20]–[22] and experimental models [23]–[25], to our knowledge no one has investigated the interaction between exercise training and AGEs formation in type 1 diabetes mellitus. Furthermore, most studies have investigated the reactivity of arteries such as aorta and mesenteric in this experimental diabetes model [26]–[31], and few studies exist evaluating femoral and coronary arteries in diabetic state [32]–[36]. Considering that diabetic patients have a 2- to 4-fold higher incidence of coronary artery disease and a ∼10-fold increased prevalence of peripheral diseases as consequence of accelerated atherogenesis [37], studies involving femoral and coronary arteries are relevant in health primary care in an attempt to prevent vascular diseases and their complications.

Our hypothesis was that 8-week exercise training would alter AGEs levels in type 1 diabetic rats ameliorating vascular responsiveness. Therefore, we investigated the effects of 8 weeks of aerobic exercise training on the vascular responses in isolated femoral and coronary artery rings from type 1 diabetic rats. To further elucidating the mechanisms involved in the vascular responses, we evaluated biochemical parameters: nitrate/nitrite (NO_x_^−^), superoxide dismutase activity (SOD), catalase activity (CAT), thiobarbituric acid reactive substances (TBARS) and CML. Protein expressions of eNOS, RAGE, cytosolic Cu/Zn-superoxide dismutase (Cu/Zn-SOD) and mitochondrial Mn-superoxide dismutase (Mn-SOD) were determined. We also evaluated the NO production and ROS generation in both tissues.

## Results

### Exercise Training Performance

The exercise training employed in our study promoted an improvement in physical performance verified in the incremental exercise test evaluated by three parameters (total time spent during the test, total distance and maximal speed). Trained animals had an increase in total time to perform the incremental exercise test (TR/DB: 13.4±0.4 min) as compared with sedentary animals (C/SD: 8.4±0.3; SD/DB: 6.0±1.1 min), approximately 90%. Similarly, the total distance performed was increased in trained group (TR/DB: 188±14 meters) as compared with sedentary groups (C/SD: 89±8; SD/DB: 53±15 meters), approximately 150%. The maximal speed was also significantly different in trained animals (TR/DB: 25±1.2 m/min) as compared with sedentary groups (C/SD: 18±1.2; SD/DB: 14±1.8 m/min), approximately 50%.

### Body Weight, Heart Weight, Food and Water Intake

**Figure 1:**
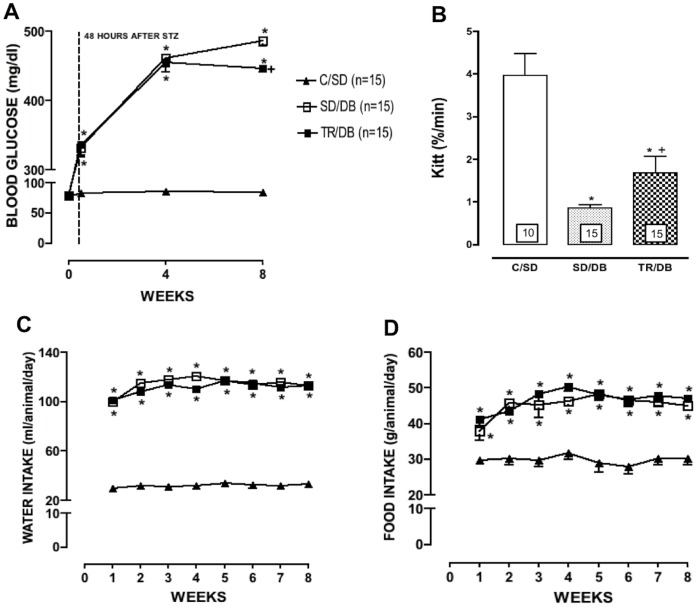
Blood glucose, insulin tolerance test, water and food intake. Blood glucose (panel A), Insulin tolerance test (Kitt, panel B) water (panel C) and food (panel D) intake. Data are mean ± SEM. The number of animals per group is indicated in the figure. *p<0.05 compared to C/SD; ^+^p<0.05 compared to SD/DB.

The body weights were similar in all groups at the initial time of the study. After 8 weeks, body weight values were significantly reduced in sedentary and trained diabetic groups as compared with control animals (Table 1). The heart weights were significantly increased in diabetic animals as compared to C/SD (Table 1). Water and food intake were increased in both sedentary and trained diabetic groups as compared with control animal (Figure 1C and 1D).

**Table 1:** Body weight, blood glucose, insulin tolerance test (Kitt), beta-ketone and heart weight in rats from control sedentary (C/SD); sedentary diabetic (SD/DB) and trained diabetic (TR/DB).

GROUPS			
	C/SD	SD/DB	TR/DB
**I-Body Weight (g)**	183±4 (15)	181±4 (15)	180±4 (15)
**F-Body Weight (g)**	400±6 (15)	208±8* (15)	213±8* (15)
**I-Blood Glucose (mg/dl)**	77±3 (15)	79±3 (15)	78±2 (15)
**STZ-Blood Glucose (mg/dl)**	82±2 (15)	332±13* (15)	336±15* (15)
**F-Blood Glucose (mg/dl)**	84±2 (15)	487±8* (15)	446±5* + (15)
**Kitt (%/min)**	4.01±0.5 (10)	0.76±0.1* (15)	1.73±0.3* + (15)
**Beta Ketone (mmol/L)**	0.8±0.06 (14)	2.2±0.3* (14)	1.8±0.4* (13)
**Heart/Body Weight (mg/g)**	2.7±0.03 (10)	3.5±0.1* (10)	3.6±0.1* (13)

Initial (I), Final (F), 48 hours after streptozotocin injection (STZ). Data are mean ± SEM. The number of animals per group is indicated in the parentheses.

* p<0.05 compared to C/SD;

+ p<0.05 compared to SD/DB.

### Blood Glucose, Beta Ketone and Insulin Tolerance Test (Kitt)

The blood glucose was similar in all groups at the initial time of the study. As expected, after 48 hours of streptozotocin (STZ) injection a significant increase in glycemia was observed in diabetic groups as compared with control animals. After 4 weeks, hyperglycemia was similar in all diabetic groups whereas exercise training for 8 weeks reduced blood glucose by 9% (Table 1 and Figure 1A). The beta ketone was increased in all diabetic groups as compared with C/SD (Table 1).

The Kitt was significantly reduced in both diabetic groups as compared with C/SD whereas exercise training partially improved the insulin sensitivity (Table 1 and Figure 1B).

### Concentration-response Curves to Vasodilators Agents

**Figure 2:**
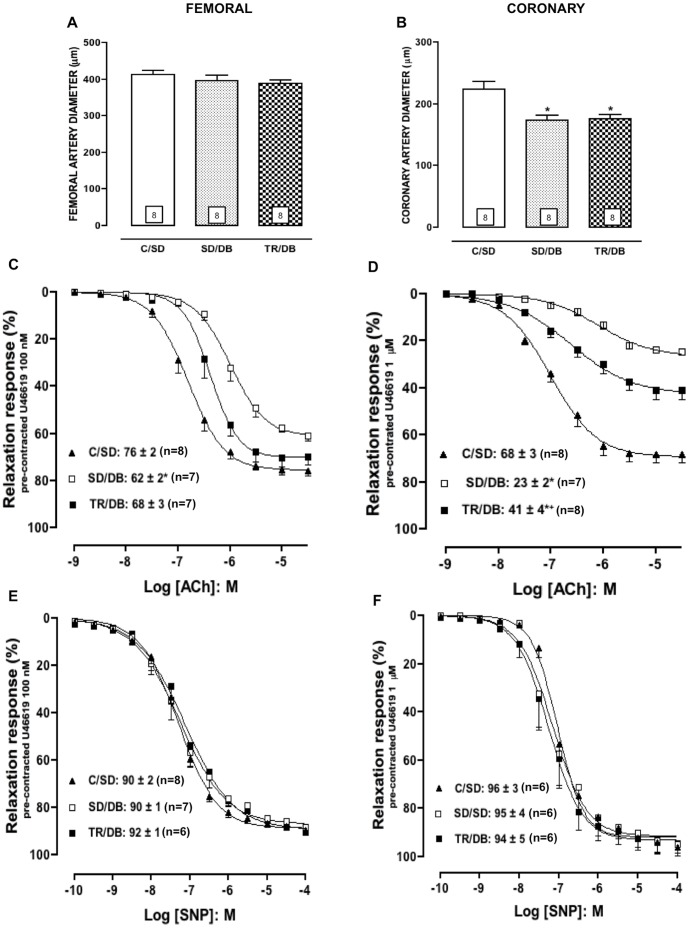
Concentration-response curves to vasodilator agents. Femoral (panel A) and coronary (panel B) artery diameters. Concentration-response curves to acetylcholine (ACh, panels C and D) and sodium nitroprusside (SNP, panels E and F), in rats femoral and coronary arteries, respectively, with intact endothelium from control sedentary (C/SD), sedentary diabetic (SD/DB) and trained diabetic (TR/DB). Maximal response values are inserted in the figure. Data are mean ± SEM. The number of animals per group is indicated in the figure. *p<0.05 compared to C/SD; ^+^p<0.05 compared to SD/DB.

Neither diabetes nor exercise training modified the femoral artery diameter in all groups (Table 2 and Figure 2A). However, in coronary artery a significant reduction was observed in diabetic groups (SD/DB and TR/DB) as compared with C/SD (Table 2 and Figure 2B).

**Table 2:** Femoral and coronary artery diameters.

GROUPS			
	C/SD	SD/DB	TR/DB
**FEMORAL DIAMETER (µm)**	413±11 (8)	395±15 (8)	388±10 (8)
**ACh**	6.81±0.09 (8)	5.98±0.10* (7)	6.37±0.06* + (7)
**SNP**	7.28±0.04 (8)	7.26±0.10 (7)	7.15±0.10 (6)
**PHE**	5.35±0.08 (6)	5.48±0.03 (6)	5.49±0.07 (6)
**U46619**	7.32±0.06 (8)	7.33±0.07 (8)	7.33±0.08 (8)
**CORONARY DIAMETER (µm)**	224±12 (8)	174±8* (8)	176±7* (8)
**ACh**	6.99±0.06 (8)	6.16±0.13* (7)	6.61±0.10* + (8)
**SNP**	7.03±0.08 (6)	7.08±0.21 (6)	7.24±0.10 (6)
**PHE**	–	–	–
**U46619**	6.69±0.15 (8)	6.71±0.12 (8)	6.59±0.13 (8)

Potency values (pEC_50_) obtained from concentration-response curves to acetylcholine (ACh), sodium nitroprusside (SNP), phenylephrine (PHE) and tromboxane mimetic 9,11-dideoxy-11α,9α-epoxy methanoprostaglandin F_2α_ (U46619) in rats femoral and coronary arteries with intact endothelium from control sedentary (C/SD); sedentary diabetic (SD/DB) and trained diabetic (TR/DB).

Potency is represented as -log of molar concentration to produce 50% of the maximal responses. Data are mean ± SEM. The number of animals per group is indicated in the parentheses.

* p<0.05 compared to C/SD;

+ p<0.05 compared to SD/DB.

The agents acetylcholine (ACh: 1 nM-30 µM) and sodium nitroprusside (SNP: 100 pM-100 µM) produced concentration-dependent relaxation in isolated femoral and coronary rings. In femoral rings a rightward shift in the concentration-response curves to ACh at the pEC_50_ level (approximately 6.3-fold) was observed in SD/DB group. The E_MAX_ values for ACh were also significantly decreased in SD/DB group as compared with C/SD. Exercise training (TR/DB) fully restored the reduction in the E_MAX_ and partially restored the pEC_50_ values for the endothelium-dependent agonist (approximately 2.5-fold). The data are summarized in Table 2 and illustrated in Figure 2C. In a similar way, in coronary rings the pEC_50_ and E_MAX_ values for ACh were significantly decreased in SD/DB group as compared with C/SD (approximately 6.7-fold) and the exercise training (TR/DB) partially restored the reduction in the E_MAX_ and the pEC_50_ values (approximately 2.3-fold). The data are summarized in Table 2 and illustrated in Figure 2D.

Regarding the concentration-responses to NO donor, SNP, no alterations were observed at the pEC_50_ levels or in E_MAX_ values in all groups for both preparation, femoral and coronary rings (Table 2, Figure 2E and 2F, respectively).

### Concentration-response Curves to Contractile Agents

**Figure 3:**
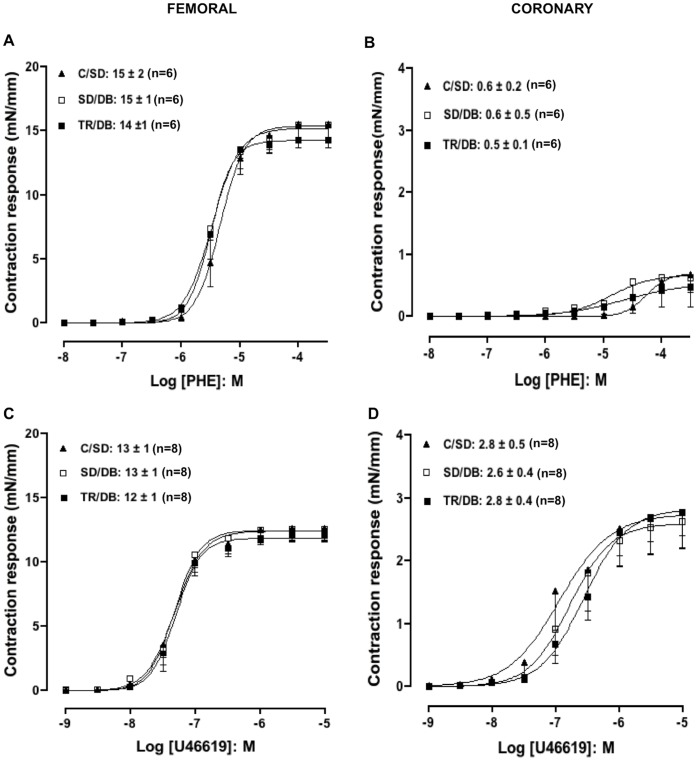
Concentration-response curves to contractile agents. Concentration-response curves to phenylephrine (PHE, panels A and B) and tromboxane mimetic 9,11-dideoxy-11α,9α-epoxy methanoprostaglandin F_2α_ (U46619, panels C and D) in rats femoral and coronary arteries, respectively, with intact endothelium from control sedentary (C/SD), sedentary diabetic (SD/DB) and trained diabetic (TR/DB). Maximal response values are inserted in the figure. Data are mean ± SEM. The number of animals per group is indicated in the figure.

The agents phenylephrine (PHE: 1 nM-300 µM) and thromboxane analog (U46619∶1 nM-10 µM) produced concentration-dependent contraction responses in isolated femoral rings in all groups, but in coronary rings, only U46619 (1 nM- 10 µM) produced contractile responses. Neither the pEC_50_ nor E_MAX_ values were modified amongst any groups in femoral (Table 2 and Figure 3A and 3C, respectively) and coronary rings (Table 2 and Figure 3B and 3D, respectively).

### Protein Expression of eNOS, RAGE, Cu/Zn-SOD and Mn-SOD in Femoral and Coronary Arteries

**Figure 4:**
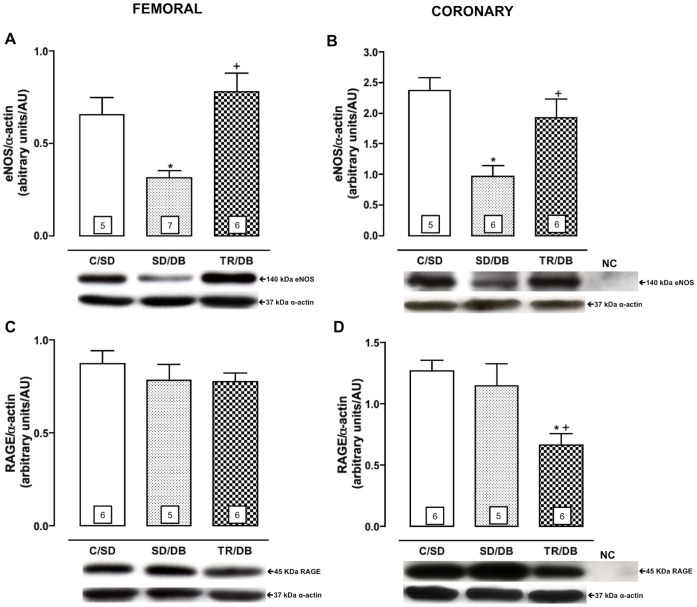
Protein expression of endothelial nitric oxide synthase (eNOS) and receptor for advanced glycation end products (RAGE). Protein expression of endothelial nitric oxide synthase (eNOS, panels A and B) and receptor for advanced glycation end products (RAGE, panels C and D) from isolated rats femoral and coronary arteries, respectively. Bottom panel representative Western Blot and top panel quantitative analysis. Data are mean ± SEM. The number of animals per group is indicated in the bars. *p<0.05 compared to C/SD and ^+^p<0.05 compared to SD/DB. Negative control (NC).

The quantification of eNOS protein expression was significantly decreased in SD/DB group, approximately 57% in femoral and 50% in coronary, as compared with control animals (C/SD). Exercise training completely restored the eNOS protein expression in femoral and coronary arteries (Figure 4, panels A and B).

The protein expression of RAGE (receptor for AGE) in femoral artery was not affected by any experimental protocols (Figure 4C). Interestingly, the RAGE expression in coronary artery was decreased, approximately 46%, in trained group (TR/DB) as compared with C/SD and SD/DB (Figure 4D).

**Figure 5:**
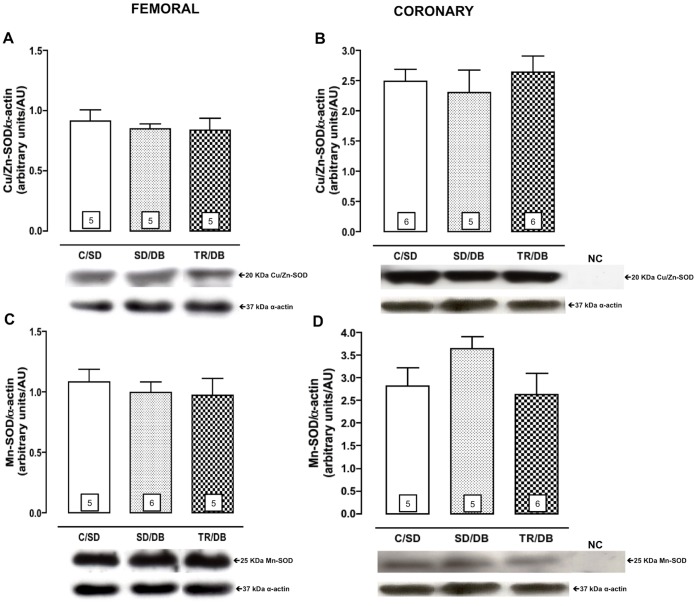
Protein expression of cytosolic Cu/Zn-superoxide dismutase (Cu/Zn-SOD) and mitochondrial Mn-superoxide dismutase (Mn-SOD). Protein expression of cytosolic Cu/Zn-superoxide dismutase (Cu/Zn-SOD, panels A and B) and mitochondrial Mn-superoxide dismutase (Mn-SOD, panels C and D) from isolated rats femoral and coronary arteries, respectively. Bottom panel representative Western Blot and top panel quantitative analysis. Data are mean ± SEM. The number of animals per group is indicated in the bars. Negative control (NC).

We further investigated the possible involvement of antioxidant enzymes in modulation the vascular responsiveness among groups. We did not observe any changes in Cu/Zn-SOD and Mn-SOD protein expressions from femoral (Figure 5 panels A and B) and coronary arteries (Figure 5 panels D and F).

### Tissues Nitric Oxide (NO) Production and Reactive Oxygen (ROS) Generation

**Figure 6:**
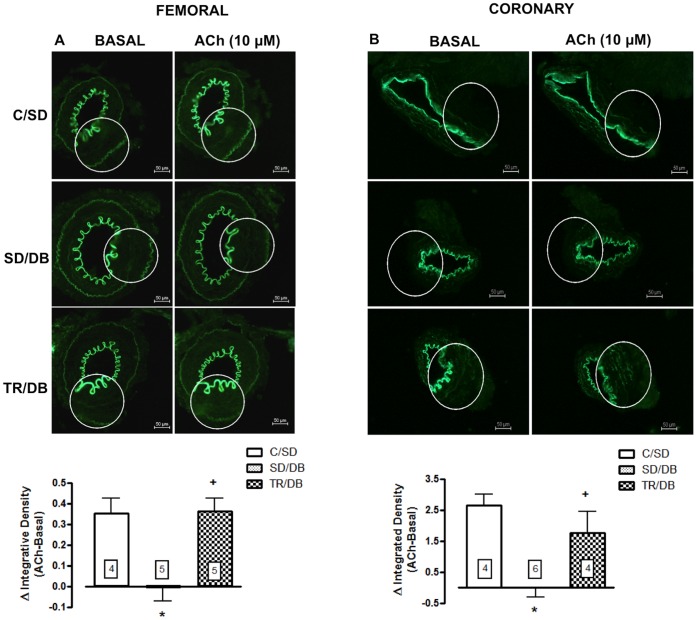
Nitric oxide (NO) production in femoral and coronary arteries. Upper panel: Representative fluorographs of DAF-2-treated sections without (BASAL) or with acetylcholine (ACh, 10 µM)-stimulation of femoral (panel A) and coronary (panel B) arteries from control sedentary (C/SD), sedentary diabetic (SD/DB) and trained diabetic (TR/DB) rats. Lower panel: Quantitative analysis of the NO production measured by DAF-2 (delta of ACh integrative density minus basal integrative density) in transverse sections of femoral (panel A) and coronary (panel B) arteries. Data are mean ± SEM. The number of animals per group is indicated in the figure. *p<0.05 compared to C/SD and ^+^p<0.05 compared to SD/DB.

**Figure 7:**
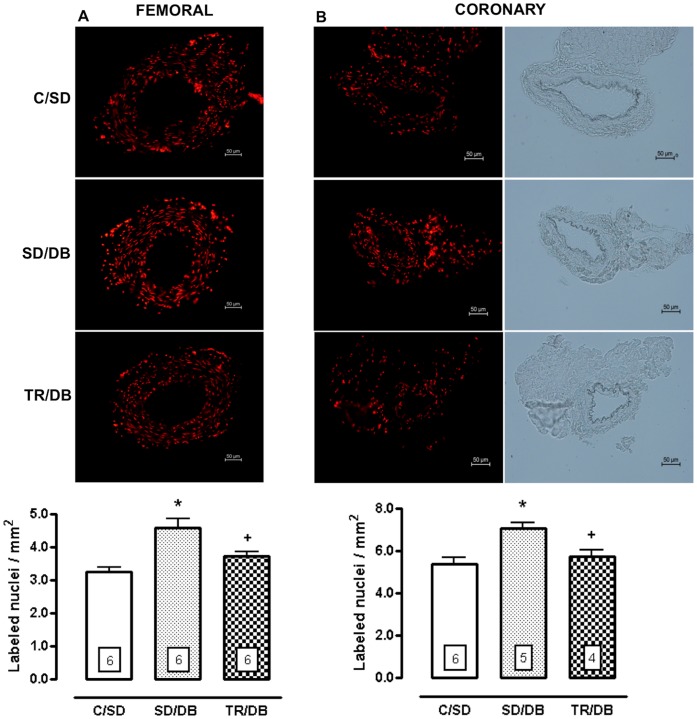
Reactive oxygen species in femoral and coronary arteries. Representative fluorographs (upper panel) and quantitative analysis (lower panel) of the ethidium-bromide-positive nuclei in transverse sections of femoral (panel A) and coronary arteries (panel B, the images with (left) and without (right) rhodamine filter) from control sedentary (C/SD), sedentary diabetic (SD/DB) and trained diabetic (TR/DB) rats. Data are mean ± SEM. The number of animals per group is indicated in the figure. *p<0.05 compared to C/SD and ^+^p<0.05 compared to SD/DB.

The NO production evoked by ACh (10 µM) was completely abrogated in SD/DB group as compared with control animals (C/SD) whereas exercise training fully restored the NO production in both femoral and coronary arteries (Figure 6, panels A and B). In additional, we observed an increase in ROS (approximately 40% and 31%, for femoral and coronary, respectively) from SD/DB animals as compared with control animals (CS/SD). Eight-week exercise training virtually abolished ROS generation induced by diabetes state in both preparations (Figure 7, panels A and B). At basal condition, NO production was not modified in both femoral and coronary arteries in all studied groups (data not shown).

### Plasma Biochemical Analysis

Plasma NO_x_^−^ levels were decreased in SD/DB by approximately 51% as compared with C/SD and exercise training TR/DB partially restored plasma NO_x_^−^ levels in diabetic animals, approximately 45% (Table 3). Interestingly, neither diabetes nor exercise training altered plasma SOD activity whereas plasma CAT activity was significantly increased in response to exercise training (TR/DB), approximately 45%, as compared with C/SD and SD/DB (Table 3).

**Table 3:** Plasma nitrate/nitrite (NO_x_^−^), superoxide dismutase activity (SOD), catalase activity (CAT), *thiobarbituric acid reactive substances* (TBARS) and N^ε^-(carboxymethyl) lysine (CML) in rats from control sedentary (C/SD); sedentary diabetic (SD/DB) and trained diabetic (TR/DB).

GROUPS			
	C/SD	SD/DB	TR/DB
**NO_x_** ^−^ **(µM)**	18.7±1.2 (11)	9.2±0.7* (10)	13.6±0.7* + (10)
**SOD (U/ml)**	30±1 (10)	29±2 (10)	30±1 (10)
**CAT (nmol/min/ml)**	94±5 (10)	88±3 (10)	133±6* + (10)
**TBARS (µM)**	5.7±0.5 (15)	16±1* (15)	10.4±0.8* + (13)
**CML (ng/ml)**	1.7±0.1 (13)	4.0±0.5* (11)	2.8±0.3* + (13)

Data are mean ± SEM. The number of animals per group is indicated in the parentheses.

* p<0.05 compared to C/SD;

+ p<0.05 compared to SD/DB.

We also analyzed whether diabetes altered oxidant status. Plasma TBARS levels were markedly increased in SD/DB, approximately 180%, as compared with C/SD animals. Exercise training partially restored this increment in TR/DB, approximately 35% (Table 3).

Diabetic state significantly increased AGEs formation measured by plasma CML levels in SD/DB group, approximately 135%, as compared with C/SD animals. This increment was attenuated by exercise training, approximately 30% (Table 3).

### Correlation Analysis

In order to investigate the relationship between plasma levels of NO_x_^−^ and CML as well as each of these parameters with the pEC_50_ values for acetylcholine obtained in the concentration-response curves in both femoral and coronary arteries, we performed a multivariate analysis. We found a negative correlation between plasma NO_x_^−^ and CML (correlation coefficient =  −0.74, p<0.05). On the other hand, there was a positive correlation between NO_x_^−^ and the pEC_50_ values evoked by ACh in both femoral (correlation coefficient = 0.70, p<0.05) and coronary (correlation coefficient = 0.58, p<0.05) arteries. Additionally, there was a negative correlation between CML and the pEC_50_ values evoked by ACh in both femoral (correlation coefficient =  −0.52, p<0.05) and coronary (correlation coefficient = −0.70, p<0.05) arteries.

## Discussion

Diabetes mellitus is a complex disease affecting several physiological systems. Cardiovascular diseases including heart attacks and strokes are the main cause of morbidity and mortality in both types of diabetes [37]. Endothelial dysfunction as consequence of hyperglycemia plays a major role in the initiation of vascular complications in diabetes [38]. In the current study, as expected, our data show a significant increase in glycemia, that was accompanied by insulin resistance, increased cardiac weight as well as a decrease in coronary artery diameters. Furthermore, a significant decrease in eNOS protein expression in both arteries was found that was accompanied by reduction in tissues NO production as well as plasma NO_x_^−^ levels. Regarding oxidative stress status and disease progression [14], [39], we found a marked increase in vascular ROS generation, plasma TBARS and CML levels in SD/DB group. When we further analyzed the relaxing responses and biomarkers altogether, our study clearly shows that the impairment of relaxing response to ACh in both femoral and coronary arteries from sedentary diabetic rats are positively associated with an imbalance in redox state caused by persistent hyperglycemia generating endothelium dysfunction with diminished NO production as well as its bioavailability to the surrounding cells in vascular tissues. Accordingly, previous studies showed similar results in aorta [40], [41] and mesenteric arteries [31]. Nevertheless, we are the first to analyze altogether NO production, AGEs formation and redox status in sedentary diabetic animals. We also demonstrated the absence of alterations in the concentration-response curves to SNP, an endothelium-independent agonist.

Exercise training is considered an important non-pharmacological tool in management of glycemia either in diabetic patients or STZ-induced diabetic animals [42]–[45]. This beneficial effect is related to activation of alternative signaling pathways in skeletal muscle involving a direct stimulation of AMP-activated protein kinase, which in turn, promotes a translocation of glucose transporters 4 (GLUT4) to the membrane, resulting in blood glucose entry into cells in an insulin-independent manner [46]. Here, we demonstrated that insulin resistance in sedentary diabetic group was partially restored by exercise training. Indeed, insulin resistance has also been described in type 1 diabetic human under insulin treatment [47]–[49] as well as in experimental models without insulin therapy [50], [51]. The possible mechanisms regarding insulin resistance have been associated with metabolic disorders characterized by reduction of fatty acid transport into mitochondria lowering its oxidation leading to increased triglycerides in skeletal muscle content [48], [52]. The reduction of total GLUT4 content and its translocation to the plasma membrane in skeletal muscle and white adipose tissue were also associated with insulin resistance in type 1 diabetes mellitus [51]. Exercise training for 8 weeks was effective in ameliorating insulin sensitivity even though the magnitude of reduction in hyperglycemia was small (9% of reduction). Indeed, evidences have shown that the benefits of exercise training per se on glycemia control in type 1 diabetes mellitus without insulin therapy is limited [53].

Regarding vascular responsiveness, physical exercise is considered a potent stimulus for NO production by activating mechanosensors present in endothelial cells that are coupled to complex biochemical signaling pathways including activation of Ras/MEK/ERK, c-Src, and PI3K/Akt pathways. [54]–[56]. Our findings show clearly that exercise training for 8 weeks prevented the reduction of eNOS protein expression and NO production in both femoral and coronary arteries from diabetic rats. We also demonstrated that exercise training fully prevented the increase in vascular ROS generation. On the other hand, plasma NO_x_^−^ levels were only partially restored in TR/DB group (approximately 45%). The possible explanation for this discrepancy between fully re-establishment of eNOS protein expression/NO production in vascular tissues and partial recovery of NO production biomarkers (NO_x_^−^ levels) could be as consequence of an increased NO inactivation. Given that, oxidative stress biomarkers measured by plasma TBARS and CML levels showed a partial reduction in TR/DB group (approximately 35 and 30%, respectively), our hypothesis is that substantial production of AGEs derived from hyperglycemia might affect endothelium function. Accordingly, AGEs could directly quench NO reducing relaxing response-dependent endothelium dependent [57], [58]. In spite of exercise training restored the balance between NO and ROS production the relaxing responses to ACh were not fully re-established in both preparations, mainly in coronary artery. The possible explanation for that is alteration of other endothelium-dependent vasodilator agents that may contribute to relaxing responses in those arteries. Indeed, it has been reported that in physiological conditions the endothelium-derived hyperpolarizing factor (EDHF) is more prominent in resistance arteries (<300 µm), such as septal coronary artery, than in conduit arteries and plays an important role in relaxing response. Of note, the balance between the endothelium-derived relaxing factors (NO, prostacyclin and EDHF) in vascular disease is not completely understood [59]–[61]. Additionally, it was described in elegant studies the importance of potassium (K^+^) channels in cardiovascular complications associated with diabetes. Specifically, it was demonstrated in coronary [62], [63], cerebral [64], aorta [65] and carotid [66] arteries that the activity and/or the response of K^+^ channels (calcium-activated K^+^ channel and ATP-sensitive K^+^ channel) are impaired in type 1 diabetic rats affecting the relaxation response evoked or not by ACh. Therefore, our study shows clearly that shear stress induced by exercise training was efficient in restoring the imbalance between NO and ROS production in non-treated diabetic rats, however, AGEs production might be deeply affected others endothelium via involved in relaxing responses in both preparations. It is noteworthy that reduction in coronary diameters observed in sedentary or trained diabetic animals might be contributing to the impairment of endothelium-dependent relaxation response as compared to femoral artery.

In contrast to previous results obtained in our laboratory, we did not find any changing in the expressions of Cu/Zn-SOD and Mn-SOD in both arteries or its plasma activity in trained diabetic animals excluding the participation of these antioxidant enzymes on the beneficial effects of exercise training on the relaxing response to ACh in type 1 diabetes mellitus. Interestingly, plasma CAT activity was significantly increased in trained groups (approximately 45%) without changing in sedentary diabetic animals. The exact mechanism behind the improvement in CAT activity after exercise training, but not in SOD protein expression or its activity is unclear for us at moment. Therefore, our data showed that the beneficial effects of exercise training on the relaxing responses to endothelium-dependent agonist in femoral and coronary arteries was directly associated with magnitude of reduction in AGE formation in diabetic animals.

Evidences have shown that AGE can act via activation of RAGE in a variety of tissues causing severe cellular damage. Furthermore, it has been reported that RAGE expression in endothelial cells, macrophages, and smooth muscle cells contributes to the pathogenesis of atherosclerosis, and particularly its acceleration in diabetes state by up-regulation of adhesion molecules as well as NOS inactivation [67]. Thus, we also analyzed RAGE expression in our study. Interestingly, RAGE expression in femoral and coronary arteries was similar in control animals and sedentary diabetic group. Accordingly, it was demonstrated no difference in RAGE protein expression in aorta from type 2 diabetic rats as compared to non-diabetic [68]. Furthermore, earliest studies have shown an up-regulation of AGEs/RAGE signaling pathway in isolated endothelial cells, but not in RAGE protein expression [69], [70]. The lack of alterations in RAGE protein expression in vascular tissues could be related to the receptor pharmacological property (a member of the immunoglobulin superfamily) which signal transduction is coupled to complex pathways involving adhesion molecules, activation of transcription factor and inflammatory mediators [71]. Thus, different from G protein-coupled receptor, mainly β-adrenergic receptors, that is susceptible to desensitization process in response to increased catecholamine levels in a short-term [72], activation of RAGE does not alter its protein expression during 8-week of diabetes state.

Interestingly, exercise training was effective in decreasing the expression of RAGE, but only in coronary artery. The mechanistic pathway by which physical training altered RAGE expression in coronary artery is not clear for us at moment.

Regarding contractile responses, both diabetes state and exercise training did not affect the reactivity of femoral artery to α1-agonist as well as to thromboxane analog. Indeed, previous studies have shown that contractile responses are not affect by exercise training in rat gastrocnemius feed artery [73] and porcine femoral [74]. Similarly, contractile responses of coronary artery rings were not modified in trained diabetic animals. Accordingly, early study has shown no difference in the contractile responses for several agonists in trained animals as compared to sedentary group [75]. Therefore, independent of blood vessels and animal species, exercise training exerts its beneficial effects primarily on endothelium-dependent responses since shear stress induced by physical exercise is a powerful stimulus in increasing NO production by endothelial cells and/or ameliorating its bioavailability to the surrounding tissues [2]. Moreover, to our knowledge only one study evaluated the contractile response in femoral artery from type 1 diabetic rats, and differently from our data, they found a leftward shift in the concentration-response curves to PHE and U46619. However, the concentration-response curves for both agonists were obtained in rings without endothelium [76].

In conclusion shear stress induced by exercise training fully restores the eNOS/NO pathway in both preparations from non-treated diabetic rats, however, a massive production of AGEs still affecting relaxing responses possibly involving other endothelium-dependent vasodilator agents, mainly in coronary artery.

## Materials and Methods

### Ethics Statement

This study was approved by the Ethical Committee for Animal Research (permit number: 1753-1) at the State University of Campinas (UNICAMP) established by the Brazilian College for Animal Experimentation (COBEA).

### Animals

Male Wistar rats, (weighing 175–195 g), were obtained from Animal Care Facility of UNICAMP and were maintained in a room at 20–21°C with normal 12 h light/dark cycle. The animals were housed in groups of two/three and had free access to water and commercial chow (Purina Co., Campinas-SP, Brazil). Animals were divided into three experimental groups: control sedentary (C/SD), sedentary diabetic (SD/DB) and trained diabetic (TR/DB).

After fasting for 12 hours, type 1 diabetes mellitus was induced by a single intraperitoneal injection of streptozotocin (STZ: 60 mg/kg, dissolved in citrate buffer pH 4.5). Control animals were injected with vehicle alone (citrate buffer). The induction of diabetes was confirmed by measuring blood glucose levels using standard test strips (Accu-Chek Performa Roche Diagnostics, Indianapolis-IN, USA) after forty-eight hours. Only rats with fasting blood glucose concentrations greater than 250 mg/dl were used in this study. Body weight, food and water intake measurements were performed weekly during all the study.

### Exercise Training

Animals were trained on a treamill designed for small animals with individual lanes (Gesan, São Paulo-SP, Brazil). The intensity of training was determined according to the plasma lactate concentration curves, representing the maximal lactate steady state for type 1 diabetic rats (Figure S1). The training program consisted of sessions of 60 min/day, 5 days/week, for 8 weeks at, 0% grade, and at a speed of 15 meters/minute (m/min). At the beginning of the training program, the duration and speed started at 10m/min for 30 min and were progressively increased to 60 min and 15m/min. One week before starting the training program, the animals were adapted to the treadmill in an attempting to minimize potential stress. Only the animals adapted were used in the present study. All the animals were trained early morning, always between 6∶00 a.m. to 8∶00 a.m.

To evaluate the effectiveness of training program, sedentary and trained rats were submitted to an acute incremental exercise testing on the treadmill during the last week of the study. The intensity of exercise was increased by 5 m/min (5–30 m/min) every 3 min at 0% grade until exhaustion. This test provided the total distance, total time and the maximal speed run for each animal.

### Blood Glucose, Beta Ketone and Insulin Tolerance Test

After 12 hours of fasting, blood samples were collected from the tail vein and glycemia was measured at baseline, 48 hours after STZ injection, 4 weeks of exercise training and at the conclusion of the study (after 48 hours of the last exercise training session). Standard test strips were used for blood glucose measurements. Blood samples were also collected for beta ketone measurements at the conclusion of the study using test strips (Optium Xceed Abbot Diabetes Care Inc, Alameda-CA, USA).

Insulin sensitivity was measured by the insulin tolerance test (ITT) and the rate constant for plasma glucose disappearance (Kitt) was calculated using the formula 0.693/biological half-life (t_1/2_). The plasma glucose t_1/2_ was calculated from the slope of the least square analysis of the plasma glucose concentration during linear phase of decline [77]. After 24 hours of the last exercise training session, rats were submitted to an ITT (2.0 U/kg), after 12 hours of fasting. Briefly, human recombinant insulin (Novolin R, Novo Nordisk, Montes Claros-MG, Brazil) was administered by intraperitoneal injection, and blood samples were collected at 0, 5, 10, 15, 20 and 30 min from tail vein and blood glucose was measured using standard test strips.

### Determination of Nitrate/nitrite (NO_x_^−^), Superoxide Dismutase Activity (SOD), Catalase Activity (CAT), *Thiobarbituric Acid Reactive Substances* (TBARS) and N^ε^-(Carboxymethyl) Lysine (CML)

After 48 hours of the last exercise training session and 12 hours of fasting, animals were anesthetized with sodium thiopental (30 mg/Kg, i.p.), arterial blood samples were collected from the abdominal aorta, centrifuged (8000 *g*, for 15 min) and the plasma supernatant was stored at −80°C.

Plasma NO_x_^−^ (µM) concentrations were measured using a commercially kit (Cayman Chemical, Ann Arbor-MI, USA). Briefly, plasma samples were ultra-filtrated through microfilter cups (Microcon Centrifugal Filter Units, 10 kDa; Millipore, Billerica-MA, USA). The NO_x_^−^ concentration of the resulting filtrate was determined based on the enzymatic conversion of nitrate to nitrite by nitrate reductase. The addition of the griess reagents converted nitrite into a deep purple azo compound and absorbance measured at 540 nm determined the nitrite concentration.

In order to analyze antioxidant status, plasma SOD (U/ml) and CAT (nmol/min/ml) activities were measured using commercially kit (Cayman Chemical, Ann Arbor-MI, USA). Briefly, SOD activity was assessed using a tetrazolium salt for detection of superoxide radicals generated by xanthine oxidase and hypoxanthine. One unit of SOD was defined as the amount of enzyme needed to exhibit 50% dismutation of the superoxide radical. The assay provided the measument of all three types of SOD and absorbance measured at 440 nm. For CAT activity the method was based on the reaction of the enzyme with methanol in the presence of an optimal concentration of hydrogen peroxide (H_2_O_2_). The formaldehyde produced was measured spectrophotometrically at 440 nm with 4-amino-3-hydrazino-5-mercapto-1,2,4-triazole as the chromogen.

The measurement of TBARS (µM) is a well-established method for screening and monitoring lipid peroxidation and it was measured using commercially available kit (Cayman Chemical, Ann Arbor-MI, USA). The malondialdehyde (MDA) -TBA adducts formed by the reaction of MDA and TBA under high temperature and acidic conditions were measured colorimetrically at 532 nm.

Plasma CML (ng/ml) concentrations were measured using commercially kit (Cell Biolabs Inc, San Diego-CA, USA). Briefly, plasma samples were used to determine the protein concentrations (Pierce BCA Protein Assay kit, Rockford-IL, USA) and samples were prepared in a concentration of 10 µg/ml. After that, the CML protein adducts present in the sample were probed with anti-CML antibody, followed by a horseradish peroxidase conjugated secondary antibody and determined by comparing with standard curve absorbance measured at 450 nm.

### Concentration Response Curves

Immediately after blood sample collection, animals were sacrificed and femoral and septal coronary arteries were isolated carefully and placed in freshly prepared ice-cold Krebs solution containing (mM): NaCl, 118; NaHCO_3_, 25; Glucose, 5.6; KCl, 4.7; KH_2_PO_4_, 1.2; MgSO_4_ 7H_2_O, 1.1; and CaCl_2_ 2H_2_O, 2.5. After the coronary isolation the heart was dried by filter-paper and then weighed. The ratio of heart to body weight was calculated.

In the sequence, the femoral and septal coronary arteries were cleaned of all adherent tissue and cut into rings of 2 mm, two wires (40 µm diameter each for femoral and 20 µm diameter each for coronary) were introduced through the lumen of segments and mounted in a small vessel myograph chamber (Danish Myo Technology, model 610M, Aarthus N, Denmark) with 5 ml Krebs solution at 37°C, pH 7.4 and continuously gassed with 95% O_2_ and 5% CO_2_ under a resting tension of 0 mN. After 15 min of equilibration period, rings were strectched to their optimal lumen diameter based on the internal circumference, wall length and wall tension using specific software for normalization (LabChart Pro-DMT Normalization Module, ADInstruments, Sydney-NSW, Australia). Data acquisition was performed using PowerLab 8/30 (LabChart 7, ADInstruments, Sydney, Australia).

Briefly, normalization was performed by distending the vessel stepwise and measuring sets of micrometer readings of force. From these measurements the internal circumference was calculated from the measured distance between the wires and the known diameter of the mounting wire. The wall length was determined using calibrated eyepiece with dissecting microscope and the wall tension was the measured force divided by the wall length. The Laplace relation was used to determine the effective pressure (P_i_) = (wall tension/(internal circumference/(2.π))). The P_i_ was an estimate of the pressure, which was necessary to extend the vessel to the measured internal circumference. The distension was stopped when the P_i_ exceeded a transmural pressure of 100 mmHg (13.3 kPa). An exponential curve was then fitted to the internal circumference pressure data and using Laplace’s equation the point on the curve corresponding to 100 mmHg was determined and denoted IC_100_. The internal circumference was set to IC_1_ = 0.9×IC_100_, since at this internal circumference the active force production of the vessel is maximal. Normalized lumen diameter (I_1_) was calculated using the equation I_1_ = IC_1_/π [78].

### Relaxation Responses to Acetylcholine and Sodium Nitroprusside

After 45 min of stabilization, rings were precontracted with KCl 80 mM and washed with Krebs to verify tissue viability. Then, femoral and coronary rings with intact endothelium were precontracted with tromboxane analog 9,11-dideoxy-11α,9α-epoxy methanoprostaglandin F_2α_ (U46619, 100 nM for femoral and 1 µM for coronary) and cumulative concentration-response curves to vasodilator agents: acetylcholine (ACh, 1 nM-30 µM) and sodium nitroprusside (SNP, 100 pM-100 µM) were obtained. Relaxing responses were plotted as percentage of the contration induced by U46619.

### Contractile Responses to Phenylephrine and Tromboxane Analog

Concentration-response curves were also obtained for contractile agents: phenylephrine (PHE, 1 nM-300 µM), in presence of beta-blocker propranolol (100 nM) or the thromboxane analog 9,11-dideoxy-11α,9α-epoxy methanoprostaglandin F_2α_ (U46619, 1 nM–10 µM). Contractile responses were plotted according to the force and length from each ring as millinewton/millimeter (mN/mm).

All the concentration-response data were fit to a logistics function in the form: E = E_MAX_/((1+ (10^c^/10^x^)^n^)+Φ), where E is the effect of above basal; E_MAX_ is the maximum response produced by the agonist; c is the logarithm of the EC_50_, the concentration of agonist that produces half-maximal response; x is the logarithm of the concentration of agonist; the exponential term, n is a curve-fitting parameter that defines the slope of the concentration response line, and Φ is the response observed in the absence of added agonist. Nonlinear regression analysis was used to determine the parameters E_MAX_, log EC_50_, and using GraphPad Prism (GraphPad Software, Prism 4, San Diego-CA, USA) with the constraint that Φ  = 0. The responses for each agonist are showen as the mean ± SEM of potency (pEC_50_) and maximal responses (E_MAX_).

### Western Blot Analysis

In order to evaluate the contribution of endothelial nitric oxide synthase (eNOS), receptor for advanced glycation end products (RAGE), cytosolic Cu/Zn-superoxide dismutase (Cu/Zn-SOD) and mitochondrial Mn-superoxide dismutase (Mn-SOD) on the vascular responses, the expression of these proteins were determined by Western blot in femoral and coronary artery tissue lysates. Frozen segments (1 segment for femoral and a pool of 2–3 segments for coronary) were homogenized in a RIPA lysing buffer (Upstate, Temecula-CA, USA) with 1 mM Na_3_VO_4_, 1 mM phenylmethylsulphonyl fluoride and protease inhibitor cocktail (2 µl/ml) (Sigma-Aldrich CO, Saint Louis-MO, USA). The tissue lysate was centrifuged (1500 g for 30 min at 4°C) and the supernatant was collected. The protein concentration was determined by BCA protein assay kit (Pierce, Rockfor-IL, USA).

Proteins from homogenized femoral artery (30 µg) were eletrophoretically (Mini-Protean II, Eletrophoresis Cell, BioRad, Hercules, CA, USA) separated by 7.5% (for eNOS) or 12% (for RAGE, Cu/Zn-SOD and Mn-SOD) SDS-PAGE. Proteins from homogenized coronary artery (30 µg) were eletrophoretically (Mini-Protean II, Eletrophoresis Cell, BioRad, Hercules, CA, USA) separated by 4–20% (Mini-Protean TGX Precast Gel, Bio-Rad, Hercules-CA, USA) SDS-PAGE.

The proteins were subsequently transferred to polyvinylidene difluoride membranes, overnight at 4°C, using a Mini Trans-Blot Cell System (Bio-Rad, Hercules-CA, USA) containing 25 mM Tris, 190 mM glycine, 20% metanol and 0.05% SDS. After blockade of nonspecific sites in Tris-buffered solution (10 mM Tris, 100 mM NaCl, and 0.1% Tween 20) with 5% nonfat dry milk, membranes were incubated overnight at 4°C with the primary antibody with a mouse monoclonal anti-eNOS (1∶1000, BD Biosciense, San Jose-CA, USA) and anti-Cu/Zn-SOD (1∶1000, Sigma-Aldrich CO, Saint Louis-MO, USA) or rabbit polyclonal anti-RAGE (1 µg/ml, Abcam, Cambridge-MA, USA) and anti-Mn-SOD (1∶1000, Axxora LLC, San Diego-CA, USA). After being washed (10 mM Tris, 100mM NaCl, and 0.1% Tween 20), membranes were incubated with secondary antibody anti-mouse (1∶2000 for eNOS, 1∶5000 for Cu/Zn-SOD, Abcam, Cambridge-MA, USA) or anti-rabbit (1∶5000 for RAGE and Mn-SOD, Amersham, Piscataway-NJ, USA) IgG antibody conjugated to horseradish peroxidase. The membranes were thoroughly washed and immunocomplexes were detected using enhanced horseradish peroxidase-luminol chemiluminescent system (ECL Plus Amersham, Piscataway-NJ, USA). Scanning densiometry was used to quantify the immunoblot signals using specific software (Scion Image, Scion Corporation, Frederick-MD, USA). The same membrane was used to determine α-actin protein expression as an internal control using a mouse monoclonal anti-α-actin (1∶30000, Abcam, Cambridge-MA, USA), and its content was used to normalize eNOS, RAGE, Cu/Zn-SOD and Mn-SOD protein expression in each sample. Negative controls for eNOS (dorsal root ganglion of rats), RAGE (bovine serum albumin), Cu/Zn-SOD (mitochondrial enriched fraction of rats gastrocnemius) and Mn-SOD (cytoplasmic fraction of rats gastrocnemius) were performed.

### Tissues Nitric Oxide (NO) Production and Measurement of Reactive Oxygen Species (ROS)

NO production was evaluated using the NO-sensitive fluorescent dye 4,5-diaminofluorescein diacetate (DAF-2, Sigma-Aldrich CO, Saint Louis-MO, USA). Femoral and coronary arteries were embedded in a freezing medium and transverse sections (10 µm) of frozen arteries were obtained as described above. Slides were equilibrated for 10 minutes in phosphate buffer (0.1 M, pH 7.4) containing CaCl2 (0.45 mM). Fresh buffer containing DAF-2 (8 µM) was topically applied to each tissue section and the slices were incubated in a light-protected humidified chamber at 37°C. After 25 minutes incubation, some sections of each artery were stimulated without (Basal) or with acetylcholine (ACh, 10 µM) for 15 minutes. At the sequence, digital images were obtained with an optical microscope (Eclipse 80i, Nikon, Japan) equipped with fluorescein filter and camera (DS-U3, Nikon, Japan), using a 20X objective. The images were analyzed with the Image J software (National Institute of Health, Bethesda-MD, USA) by the integrative density of the fluorescence observed in the artery in relation to the background staining in sections with and without ACh-stimulation. The results were expressed as the delta of ACh-stimulation integrative density minus basal integrative density.

The oxidative fluorescent dye hydroethidine (Invitrogen, Grand Island-NY, USA) was used to evaluate *in situ* ROS generation, as previously described [79]. Hydroethidine permeates cells freely and in the presence of superoxide anions, is oxidized to ethidium bromide, which is trapped by intercalation into DNA. Femoral and coronary arteries were embedded in a freezing medium and transverse sections (10 µm) of frozen arteries were obtained on a cryostat, collected on glass slides and equilibrated for 10 minutes in Hanks solution (in mM: 1.6 CaCl_2_; 1.0 MgSO_4_; 145.0 NaCl; 5.0 KCl; 0.5 NaH_2_PO_4;_ 10.0 dextrose; 10.0 HEPES; pH 7.4) at 37°C. Fresh Hanks solution containing hydroethidine (2 µM) was topically applied to each tissue section and the slices were incubated in a light-protected humidified chamber at 37°C for 30 minutes. Negative control sections received the same volume of Hanks solution but in the absence of hydroethidine. Images were obtained with an optical microscope (Eclipse 80i, Nikon, Japan) equipped with filter to rhodamine and camera (DS-U3, Nikon, Japan), using a 20× objective. The number of nuclei labeled with ethidium bromide (EB-positive nuclei) along vascular wall was automatically counted using Image J software (National Institute of Health, Bethesda-MD, USA) and expressed as labeled nuclei/mm^2^.

In the specific condition of the coronary tissue as well as the material was collected surrounded by cardiac muscle, to quantify the EB-positive nuclei into the coronary artery, without misinterpretation, first the images were obtained without rhodamine filter to limit the artery into the cardiac muscle. After that, the number of nuclei labeled with ethidium bromide (EB-positive nuclei) along vascular wall was automatically counted using Image J software (National Institute of Health, Bethesda-MD, USA) and expressed as labeled nuclei/mm^2^.

### Statistical Analysis

Data are expressed as mean ± SEM of *n* experiments. One-way ANOVA followed by a Tukey’s test was performed using Instat Software (GraphPad Software, San Diego-CA, USA). Values of *p<*0.05 were considered statistically significant. Analysis for correlations was performed using Person’s approach. A multiple regression analysis was done using a general linear model performed using Statistica 7.0 Software (Statsoft, Tulsa-OK, USA). Values of *p<*0.05 were considered statistically significant.

### Drugs

Acetylcholine chloride, DL-Propranolol hydrochloride, sodium nitroprusside dihydrate, streptozotocin, phenylephrine hydrochloride, tromboxane analog 9,11-dideoxy-11α,9α-epoxy methanoprostaglandin F_2α_ were purchased from Sigma-Aldrich CO. (Saint Louis-MO, USA). TBS Tissue freezing medium was from Triagle Biochemical Sciences (Durhan-NC, USA). Hydroethidine was purchased from Invitrogen (Grand Island-NY, USA) and 4,5-diaminofluorescein diacetate was from Sigma-Aldrich CO (Saint Louis-MO, USA).

## Supporting Information

Figure S1 **Determination of maximal lactate steady state in type 1 diabetic rats. The animals presented a stabilization of blood lactate at 10 m/min (3.3±0.3 mmol/L) and 15 m/min (3.6±0.3 mmol/L).** There was a progressive increase in blood lactate with higher speed 20 m/min (6.0 mmol/L). Data are mean ± SEM for 12 animals in each speed.
(TIFF)
