# Supplementary figures and images for: Interaction between Advanced Glycation End Products Formation and Vascular Responses in Femoral and Coronary Arteries from Exercised Diabetic Rats
Source: PLoS One. 2012 Dec 28;7(12):e53318. doi: 10.1371/journal.pone.0053318 (PMC3532341; doi:10.1371/journal.pone.0053318)

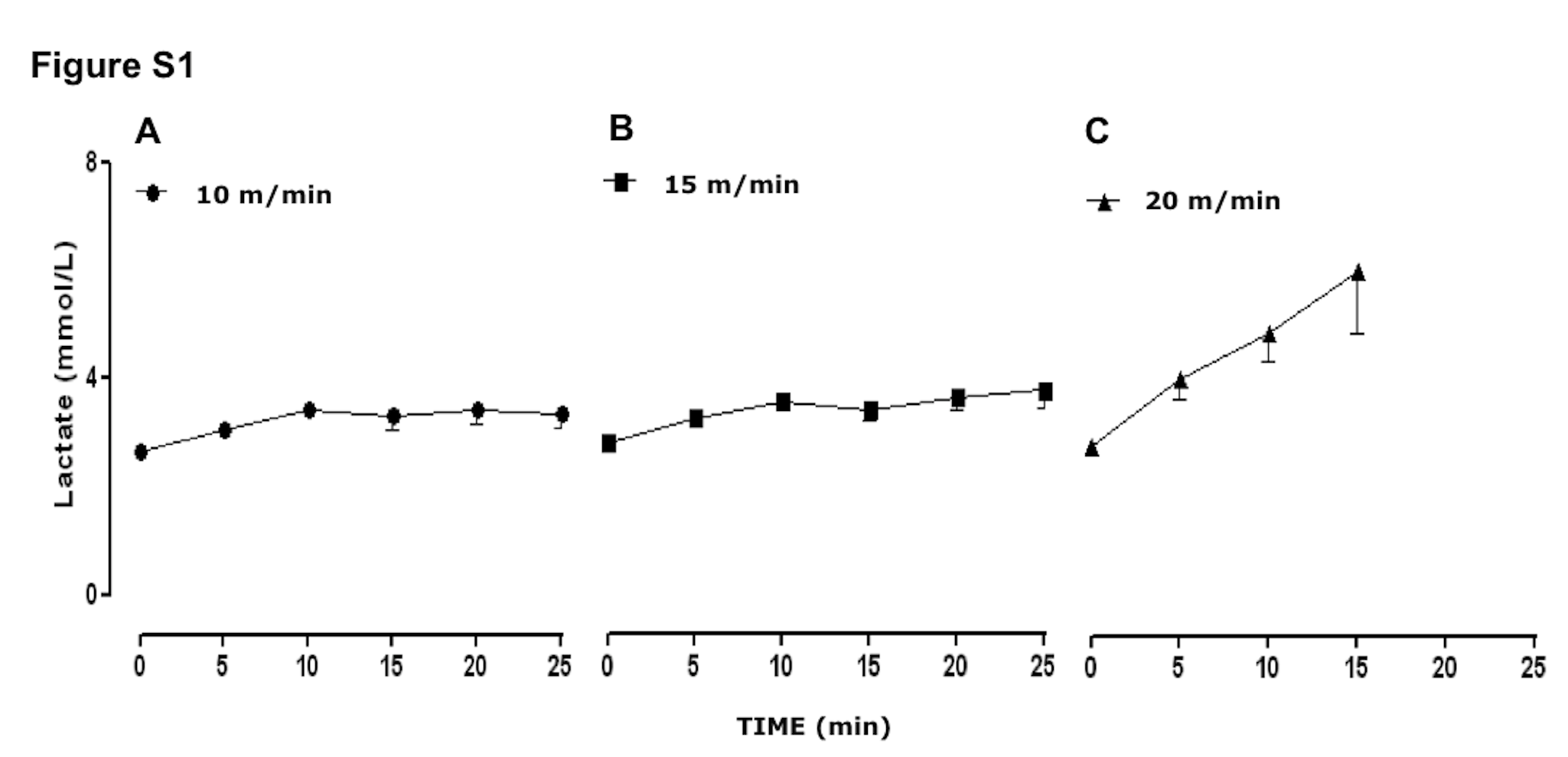

Supplement: Figure S1 — Determination of maximal lactate steady state in type 1 diabetic rats. The animals presented a stabilization of blood lactate at 10 m/min (3.3±0.3 mmol/L) and 15 m/min (3.6±0.3 mmol/L). There was a progressive increase in blood lactate with higher speed 20 m/min (6.0 mmol/L). Data are mean ± SEM for 12 animals in each speed. (TIFF) [file pone.0053318.s001.tif]
